# A descriptive qualitative study of pregnancy experiences, care and decision-making amongst South African adolescent girls and young women

**DOI:** 10.3389/frph.2026.1744098

**Published:** 2026-01-30

**Authors:** Lynné Kapiera, Kate Bergh, Kim Jonas, Catherine Mathews, Colleen Wagner, Zoe Duby

**Affiliations:** 1 Health Systems Research Unit, South African Medical Research Council, Cape Town, South Africa; 2 Department of Psychology, University of Cape Town, Cape Town, South Africa; 3 School of Public Health, University of Cape Town, Cape Town, South Africa; 4 Networking HIV/AIDS Community of Southern Africa (NACOSA), Cape Town, South Africa

**Keywords:** adolescent girls, decision-making, descriptive qualitative research, experience, pregnancy, pregnancy-related services, support

## Abstract

**Background:**

Pregnancy among adolescent girls and young women (AGYW) remains a key public health concern, shaped by wider socio-cultural and structural factors. Although global birth rates among adolescent girls are declining, South Africa's rates continue to rise. This study examined AGYW's attitudes, experiences with pregnancy and antenatal care, and views on accessing related services.

**Methods:**

The study employed a descriptive qualitative design, using in-depth interviews to explore the experiences of AGYW aged 15–24 years from seven South African provinces, regardless of pregnancy history. Participants were selected from the HERStory 3 survey database and interviewed remotely by telephone, with sixty-eight participants. Interviews were transcribed and translated into English. Data analysis followed an integrated, cyclical thematic approach that identified themes through deductive and inductive methods to identify and define themes, patterns, and relationships.

**Results:**

The study identified four main themes: support during pregnancy, mental health, health service experiences, and social or cultural factors affecting pregnancy experiences. Family, particularly parents, played a major role in the pregnancy experiences of AGYW, but limited partner communication and difficulty confiding in families often weakened support networks. Participants reported issues, like premature birth and miscarriage, highlighting a need for postpartum care. Experiences with health services were mixed, with some unaware of available programs. Decisions about abortion involved financial, educational, health, and moral considerations.

**Conclusions:**

This study examined AGYW's experiences of pregnancy and pregnancy-related services. Findings suggest that SRH services for AGYW in high-risk communities can be improved by training support professionals to be more sensitive and non-judgmental.

## 1 Introduction

The experience of pregnancy among adolescent girls and young women (AGYW) constitutes a critical aspect of sexual and reproductive health (SRH), intricately linked to wider socio-cultural and structural factors (1). Adolescent pregnancy, defined as pregnancy in females 10–19 years in age (2), can have serious health complications for the pregnant female and limit their socioeconomic opportunities (3). Pregnancy among AGYW is influenced by factors such as early age, intentionality in becoming pregnant, availability of support systems, and healthcare accessibility and acceptability (4–7). Teenagers with limited maternal health services may miss crucial HIV testing, treatment, and early detection of complications (8). Pregnant AGYW tend to have lower health literacy than older pregnant women, and little knowledge of what to expect during and after pregnancy (9). Additionally, negative psychosocial experiences of pregnancy in AGYW involve changes to quality of life, stigma, and mental health issues such as depression, anxiety, mood disorders, and post-traumatic stress disorder (10). However, timely, high-quality antenatal care (ANC) enables early screening and management of risk factors like hypertension, tuberculosis, HIV, respiratory issues, high blood sugar, anemia, eclampsia, and mental health in pregnant AGYW (9, 11).

The World Health Organisation (12) reported a global decrease in adolescent birth rates from 64.5 to 41.3 births per 1000 women from 2000 to 2023, but the rate in South Africa continued to rise annually (13). In 2019, adolescents in South Africa delivered 129,223 births in public health facilities; KwaZulu-Natal recorded the highest share at 28%, while Free State and Northern Cape recorded less than 5% (14). These figures demonstrate a substantial number of pregnancies and live births among South African adolescents, but do not account for young women aged 20–24 years old, indicating both a gap in the existing data and a need to understand the circumstances behind these occurrences.

Pregnancy studies have often overlooked the intricate interplay of factors, such as socioeconomic status, low self-esteem, vulnerability to sexual abuse, and the influence of peers on decisions to engage in transactional sex, making understanding teenage pregnancy more complex and requiring a deeper understanding of these dynamics (3). Self-esteem can be undermined by socioeconomic disadvantages, and low self-esteem makes one more vulnerable to abuse or poor decision-making. Peer pressure can normalise risk behaviours or accentuate the effects of financial instability. Since these elements interact and overlap—rather than function independently—comprehending and managing AGYW pregnancy is difficult and calls for nuance, compassion, and a multifaceted approach.

### 1.1 Study rationale and aims

Although there is evidence on some of the negative biological and social outcomes of early pregnancy, limited research exists on pregnancy experiences, healthcare interactions, and decision-making among South African AGYW, with previous studies excluding adolescents under 16 years old (8), only including one or two languages (8), focused on healthcare facilities in urban settings in one city (9), excluded perspectives on ANC between adults and adolescents (9) and excluded perspectives on how family, peer and partner support can address social and economic changes experienced by AGYW during and after pregnancy (15). The study fills literature gaps by involving adolescents aged 15–17, multiple languages, diverse study sites, and blending the views and experiences of adolescent girls and young women.

Our study used qualitative methods to explore the views and experience of pregnancy and related services among AGYW across South Africa. Qualitative inquiry places a priority on people's lived experience and the meanings they ascribe to their experiences (16). Hence, this study followed a descriptive qualitative approach to examine views of pregnancy among AGYW, to document AGYW experiences of pregnancy and pregnancy-related services, and to understand views on the accessibility of pregnancy-related services for AGYW in their community.

These findings build upon and add to existing literature (8, 9, 15, 17, 18) by exploring the perspective of AGYW in sites where interventions for HIV and SRH services took place.

## 2 Method

### 2.1 Study design

This paper presents analysis from the HERStory3 qualitative study, a component of a mixed-methods evaluation of a combination SRH intervention for AGYW in South Africa (6, 17, 19–22). Following the example of previous studies on pregnancy (23–26), the findings discussed in this paper employed a qualitative descriptive design to comprehend the experiences and views of pregnancy, care, and decision-making among AGYW. The descriptive qualitative design offered an in-depth, clear characterisation and comprehensive understanding of a phenomenon (27). Without imposing pre-existing notions, it aided in gathering and analysing data to gain insights into the participants' perspectives and experiences on pregnancy and care, proving appropriate for learning about this topic in depth.

### 2.2 Sampling and recruitment

The sample comprised AGYW aged between 15 and 24 years who had taken part in the HERStory3 quantitative household survey conducted in eleven subdistricts across seven provinces, where the combination intervention (28) was being implemented: AbaQulusi and uMhlathuze in KwaZulu-Natal province (KZN), Mbombela and Govan Mbeki in Mpumalanga province (MP), Nelson Mandela Bay and Nyandeni in Eastern Cape province (EC), Dihlabeng and Setsoto in Free State province (FS), Tshwane 1 in Gauteng province (GP), Rustenburg in North West province (NW), and Klipfontein in the Western Cape province (WC). If they fulfilled all the requirements listed in the selection criteria (Table 1), residents of the designated study areas were eligible to participate in the survey and the qualitative interviews that followed.

**Table 1:** Selection criteria for eligible study participants of the quantitative and qualitative study.

Inclusion criteria	Exclusion criteria
Female household resident aged 15–24 years	Cognitive or mental challenges (based on the assessment of the participant's ability to comprehend the study information provided)
Female household resident <18 years of aged who had consented and who parent, guardian, caregiver, or household representative had consented	Deaf or mute
Residing in the selected household	Unable to speak English, isiZulu, isiXhosa, seSotho, Setswana, Xitsonga, isiSwati, Sepedi, Afrikaans, or isiNdebele
Willing to provide written informed consent	Not available for participation between 8 AM. and 9 PM
Willing to participate in the study	Female household resident <18 years of aged who had consented but whose parent, guardian, caregiver, or household representative had not consented
Willing to undergo study procedures	
Willing to provide a biological sample for quantitative study	

The HERStory3 quantitative survey database was used to purposively select participants for the qualitative interviews. A final question in the HERStory 3 quantitative survey asked participants if they would be willing to participate in an in-depth interview (IDI). Their cellphone number was entered into a database if the participant consented to being contacted. A referral list was generated from those survey participants who indicated interest in being interviewed, with each AGYW assigned a unique participant identification (PID) determined by the age group and the subdistrict from which she was referred.

Trained female interviewers were assigned participants based on language proficiency and primary language spoken in each subdistrict (isiZulu, Sesotho, Setswana, Afrikaans, isiXhosa or English). Using a screening script that included information about the study, interviewers contacted the AGYW listed in the enrolment log to invite them to participate. Interviewers noted each participant's willingness to engage in an interview and whether or not a potential participant was successfully reached. Before scheduling the interview, those AGYW who were successfully reached and agreed to participate went through a consent process.

### 2.3 Data collection

The data was gathered between March and May 2024 through telephonic in-depth interviews conducted both serially and once-off. Telephonic interviews were cost-effective, flexible, and time-efficient for the participants, which was appropriate for remote interviews. Using Microsoft Teams software, interviews were audio recorded with participants' permission and lasted approximately an hour. The South African Medical Research Council (SAMRC), in collaboration with intervention implementers and partners, developed the topic guide used during the interviews.

### 2.4 Data analysis

The raw data consisted of English transcripts translated from interview audio recordings. A thematic analysis was chosen to transform raw data by searching, evaluating, recognising, coding, mapping, exploring, and describing patterns, themes and categories to interpret their underlying meaning. Conducted by LK and ZD, the analysis followed an iterative, cyclical process in which data collection and analysis occurred simultaneously. Themes were refined continuously (29), allowing for a comprehensive and nuanced understanding of the material, which was suitable for our study.

ZD initially developed a set of deductive code types based on the topics included in the interview guide. LK and ZD then read and coded the transcripts, expanding and refining the codebook through inductive analysis and discussion. The analysis process evolved iteratively through a deductive and inductive process reflecting the study's key objectives by LK under the guidance of ZD. This collaborative process produced clear code definitions and examples. LK identified sub-themes related to pregnancy experiences and views, and together LK and ZD further refined and expanded thematic areas. LK also used pattern themes to explore relationships within the data, achieving greater conceptual depth. The iterative process of reading and identifying emergent themes helped to interpret the data. KB, KJ, CM and CW reviewed the themes with LK and ZD and worked towards consensus, ensuring interpretation aligned with the data and study objectives.

### 2.5 Ethical considerations

Ethical approval for the study was obtained from the Human Ethics Research Committee at the South African Medical Research Council (EC027-8/2023). Research ethics-trained interviewers obtained informed consent from prospective participants using English or local language consent information sheets. Participants were informed about the study, given the chance to ask questions, and provided anonymised consent, which was audio recorded. Verbal consent was obtained from parents or caregivers for participants younger than 18 years old.

Participants were asked to be in a private space before the start of the interview to ensure privacy and confidentiality of the conversation. They had the option to end their participation and decline to answer questions at any time throughout the interview. A voluntary withdrawal was considered if a participant hung up the phone before the interview finished. Participants were offered the option to resume the interview if they phoned and said that the hang-up was an error. After completion, participants received a redeemable voucher worth ZAR 200.00 (approximately US$ 11.00) as reimbursement.

The study recorded no significant adverse events, no potential victims of emotional or sexual abuse, and no vulnerable individuals needing referral, such as HIV-positive individuals or children without adult supervision or support. Referral systems were in place for any participants requiring mental health support or social protection.

## 3 Findings

In the HERStory 3 quantitative study, 2,638 AGYW in the intervention sites participated in the survey; 348 AGYW (Table 2) were contacted and screened for participation in telephonic qualitative interviews.

**Table 2:** Total number of adolescent girls and young women (AGYW) screened for participation in telephonic qualitative interviews conducted in March, April and May 2024.

Screening activities	March	April	May	Totals
AGYW called	90	218	40	348
AGYW successfully contacted	33	62	17	112
AGYW contacted and declined participation	5	7	2	14
AGYW successfully screened: Eligible and willing to participate	24	58	9	91
AGYW successfully consented	15	50	8	73
AGYW successfully interviewed (inclusive of once-off and serial interviews)	15	45	8	**68**

Bold indicates the total number of participants successfully interviewed between March and May 2024.

Out of the primary qualitative interviews, sixty-eight AGYW answered questions about pregnancy experiences, opinions and experiences regarding healthcare accessibility, and opinions regarding abortion.

When comparing the number of participants with and without pregnancy experience (Table 3), young women aged 18–24 years (*N* = 53/68) made up the biggest cohort of participants, followed by adolescent girls aged 15–17 years (*N* = 15/68). A total of 22 participants had prior pregnancy experience; among the 53 young women aged 18–24, a total of 21 reported having had pregnancy experiences; among adolescent girls 15–17 years, only one participant (*N* = 1/15) reported having had prior pregnancy experiences. In contrast, 46 of the 68 participants in this study reported having no prior experience with being pregnant; among the 53 young women aged 18–24, a total of 32 reported they had never had a pregnancy before; among adolescent girls 15–17 years, all except one participant (*N* = 14/15) had never experienced being pregnant.

**Table 3:** Comparison of the number of participants with and without pregnancy experience per age category in each province.

Province	Number of participants with pregnancy experience		Number of participants without pregnancy experience	
	15–17 years	18–24 years	15–17 years	18–24 years
KwaZulu Natal	0	2	0	4
Mpumalanga	1	8	0	8
Eastern Cape	0	0	2	2
Free State	0	1	4	6
Gauteng	0	3	2	3
North West	0	1	3	5
Western Cape	0	6	3	4
Total	**1**	**21**	**14**	**32**
	**22**		**46**	

Bold indicates the total number of participants who had experienced pregnancy and who had not experienced pregnancy in both 15-17 years and 17-24 years age categories.

Participants who personally experienced a pregnancy described their experiences of being pregnant and shared their views and perceptions of pregnancy-related services in their communities. Participants who had not experienced a pregnancy largely shared their views and opinions on the sociocultural contexts of pregnancy and the accessibility of pregnancy-related services in their communities.

The four main themes that emerged related to the following common discussion points: support during pregnancy, mental health and pregnancy, health services experienced during pregnancy, and social and cultural contexts affecting pregnancy experiences. Findings are presented below alongside quotations from English transcripts. Participants' details (site, age group) are provided in brackets after each quotation.

### 3.1 Support during pregnancy

Participants' experiences with the different forms of support that they received during their pregnancies emerged in the first main theme. The various kinds of support enabled them to handle the numerous issues and changes that they may have encountered during pregnancy. Some participants who experienced being pregnant stated that they mainly received support from their family and partners, whereas other AGYW revealed how a lack of support affected their pregnancy journey.

#### 3.1.1 Support from parents/caregivers/family

Some participants mentioned that their experience of pregnancies were positively influenced by family support, particularly from a parent. AGYW who experienced pregnancy primarily discussed the material and emotional support their mothers provided:

Yes. There is someone I was talking with at home…My mother used to help me with everything, I got everything I needed from her. (Gert Sibande, MP—18–24 years)

Furthermore, a few participants, particularly those in the younger age group, said they would ask their mother for assistance and support if they thought they might be pregnant:

[laughter] I would be scared…I would seek help…From my mom. (Thabo Mofutsanyana, FS—15–17 years)

A few participants said that if they tested positive for pregnancy, they would go to an older sibling for support and advice, fearing the reaction of other family members:

I don't want to lie, I wouldn't know what to do…I would first talk to my elder sister…Because family (members) kill each other…I would talk to my sister first…I will wait for her intelligent reply and advice. (Ehlanzeni, MP—18–24 years)

#### 3.1.2 Support from partners

A few AGYW stated that if a pregnancy test came back positive, they would notify the father of their unborn child:

I would start…by, uh, telling my boyfriend that I'm pregnant. (Thabo Mofutsanyana, FS—18–24 years)

In some cases, such as the following example, AGYW who experienced pregnancy found it easier to disclose their pregnancy to their partner than their family:

The first time getting pregnant is difficult; you do not know who to tell, what to do. Like me, for example, when I got pregnant, I only told my boyfriend. At home they just saw my tummy grow. It's then they asked that “Are you pregnant?” then I said “Yes I am pregnant”, but it was them who saw it. When I found out I was pregnant, I did not tell anyone at home because it was hard for me to tell them. (Bojanala, NW—18–24 years)

#### 3.1.3 Impact of lack of support during pregnancy

Some young women found it challenging to decide who to inform about their first experience with pregnancy. A few AGYW mentioned that the reason for the difficulty in disclosing their pregnancies to their parents was out of fear of disappointing them:

Most of the girls say they are afraid to talk to their parents and may only be able to talk to them when they are pregnant, my opinion on that…They might feel scared because they feel that they have disappointed their parents and it is not easy telling your parents that you are bring a human to this world. (Zululand, KZN—18–24 years)

Another reason why AGYW fear disclosing their pregnancies to their parents was the threat of parental rejection, leading many to opt for abortions:

What can I say. Girls are afraid to open up about their pregnancy because parents always say they will kick us out if we fall pregnant…Hence why they choose to have abortions. (Ehlanzeni, MP—18–24 years).

Lack of support from partners also had an impact on AGYW who experienced pregnancy. As one participant expressed, the dynamics in her relationship shifted when she was pregnant, where her partner made excuses when she asked for financial assistance, leading to acceptance that her requests may not be met:

When I was pregnant, you crave for things you sometimes can't get, boys change when you are pregnant. Whenever I'll ask for money, there will be excuses, so at times I would accept that what I don't have. (Zululand, KZN—18–24 years)

### 3.2 Mental health and pregnancy

AGYW are still developing in their bodies and emotions, and the status and progression of their pregnancy can compound the difficulties they face during this period of adolescence and young adulthood. Participants shared their struggles with their mental health during significantly difficult pregnancy and birth experiences, and views on AGYW pregnancy stigma in their communities. In an example of a difficult birth in which AGYW received critical postpartum support, one participant described how she gave birth to both of her children prematurely:

Umm! A very rough road I can tell you, she (my baby) was born at seven months. I was 3 times in labour with my daughter six months…and in seven months she was born…I stayed in hospital for seven days with her, but all I can tell you it was depressing at the time. But she's a miracle…Because you have one foot in the grave and one foot outside the grave…So, you don't know actually what will happen during your pregnancy or even after your pregnancy. (They) gave me the best services I can say. (Cape Town, WC—18–24 years)

Another participant experienced a miscarriage, which led to a decline in her health. However, despite the challenges, she was aided by a social worker at her school to express how she felt in order to heal from that traumatic event:

I had miscarriage that year…It's been a hard year for me, so my health deteriorated, but I've managed to cope with it until now…A social worker was helping me actually at my school…So, I would go to her to…that lady she would actually help me…It was basically like she would just tell me that I should speak about my feelings. (Cape Town, WC—18–24 years)

One participant discussed the emotional effects of a stillbirth on her life and the support she received from a social worker, family and friends after experiencing symptoms of depression, providing another illustration of the mental health effects of a challenging pregnancy:

I've experienced a stillborn…it's almost like a miscarriage. Not exactly a miscarriage because I still gave normal birth…Miscarriage is when you bleed out just blood, but I gave normal birth, so it was a stillborn…After that, I've been seeing social worker also regarding that case…I was down for a couple of months, but after that I was back on my feet. I didn't feel as, as much as I felt in the first two months…It was very difficult…my family members, peers, I had a lot of friends really concerned and they were there for me. (Cape Town, WC—18–24 years).

Participants also discussed stigma that pregnant AGYW faced in their communities. Some participants mentioned that pregnant AGYW hesitate to seek healthcare services for fear of judgement, particularly from elders, regarding early parenthood:

Some are scared of getting judged by the community, you know how elderly people are when you have a baby when you are so young, so I would tell like have like go for it and just get uum! services at the clinic and raise a baby. (Tshwane 1, GAU—AGYW 18–24 years).

Considering the fear of judgement, some participants discussed how pregnant AGYW would delay ANC initiation until complications in their pregnancy arise, choosing to hide their pregnancy to avoid judgement, or only visit clinics when visibly advanced in their pregnancy:

Some get the help and others don't, others want to go to the clinic when the belly is big, whereas there should go when their belly is still small. They only want to go when they experience complications…They hide their pregnancy with jerseys because people tent to judge them. (Ehlanzeni, MP—18–24 years)

### 3.3 Health services experienced during pregnancy

Antenatal care (ANC) is a crucial health care service provided to expectant women that ensures their health and the health of their unborn babies during pregnancy. However, a variety of factors can influence AGYW's likelihood of accessing these services, as well as their frequency of attendance at scheduled appointments. Participants discussed their experiences with healthcare services during their pregnancy journey and shared their perspectives on healthcare accessibility in their communities.

#### 3.3.1 Health services access during pregnancy

Regarding their personal health and accessing ANC services, a few participants who experienced pregnancy talked about how their pregnancy journey was relatively easy, and they managed to access the services they needed from the local clinics:

I did not have any challenges during my pregnancy; I was well and healthy…I went to the clinic…They ran various tests…because doctors are very expensive…and the clinic is free. (Gert Sibande, MP—AGYW 18–24 years)

A few individuals who experienced pregnancy recounted good care from healthcare workers (HCW) and plenty of emotional support in pregnancy services for expectant AGYW at government facilities:

I went to the clinic…They treated me fairly…Although they were slow but the treatment was good…(They) were women…They had patience and they were communicating well with me. They made you feel at home. (Ehlanzeni, MP—AGYW 18–24 years)

Conversely, very few participants who experienced pregnancy reported unfavourable experiences with ANC. One adolescent participant detailed mistreatment by certain clinic staff during routine ANC, citing a lack of emotional support because she was considered too young to be pregnant:

It was not easy because I was too young to have a baby… It depended on the people that were giving me help, some were shouting at me, (asking) why I got pregnant at this age and I felt bad. (Ehlanzeni, MP—15–17 years).

Another participant shared her experience, suggesting that complaints from patients—especially pregnant AGYW—who suffered mistreatment from clinic staff were not taken seriously:

there was a nurse the whole ward complained about, she would turn a blind eye to everyone who needed help…A lady that cooks in the hospital told us that they will not read the complaints we wrote. No one will pay notice and we all will be discharged by the time these complaints are read. (Zululand, KZN—18–24 years)

A few participants who experienced pregnancy expressed sadness about being treated poorly at the clinic, where they were often sent away without consideration for their time or travel expenses. Instances included being told to return on a different day if they arrived late, or waiting for staff, resulting in many being turned away at closing time:

It was sad to us who were pregnant because when you entered the room without knocking, we would be told to go back to where we came from…Even if I would arrive late, you would be told return back home and come back anther day…only to find that they are doing that to go eat their lunch. Or when they go for lunch, we will wait for them, and if the clock hits four it will be closed…And they will take 6 or 7 of us, they rest are told to come back another time. They would not empathize about the money that we used to come to the clinic. (Zululand, KZN—18–24 years)

#### 3.3.2 Views on accessibility of healthcare services

The operations and organisation of the healthcare facility pregnant AGYW selected had an impact on service accessibility. The relationship between AGYW, the clinic staff, and the organisation of services offered at the facility demonstrated this notion. One participant thought that it was easy for pregnant AGYW to get healthcare services they needed, considering that the clinics were situated in closer proximity to their community and they were easily referred to ANC services:

I think it's easy…When you go to the clinic, they no longer make a fuss…when you tell them what you came for, they just automatically direct you to the right place…It's because those services are available and nowadays clinics are even closer. (Tshwane 1, GAU—18–24 years)

In contrast, as one participant observed, it would be difficult for AGYW to access healthcare services if they had to spend more money and time on travelling to appointments at clinics that were further from their place of residence:

No, not really…Sometimes it will be maybe money, finance…To get services from the clinic, which is what I do when I go to the clinic, I travel with a taxi to get to my nearest clinic. (Bojanala, NW—18–24 years)

Some participants noted that pregnant AGYW could attend multiple programmes, not just the clinic, despite the financial implications of attending ANC to receive the resources and care their require:

It is very easy because there are lot of young children who are currently pregnant…there is a lot of programmes and there is a lot of people who are also encouraging (Cape Town, WC—18–24 years)

Some participants mentioned that many pregnant AGYW fail to seek necessary prenatal care either because of a fear of criticism for becoming pregnant at a young age, which discourages them from attending ANC:

I don't think, because nowadays people do not use facilities for their prenatal visits because they are lazy…It could be they are criticized because they were pregnant at a very young age. Hence that is why they are afraid of going to clinics. (Zululand, KZN—18–24 years)

### 3.4 Social and cultural contexts affecting pregnancy experiences

AGYW who become pregnant often encounter stigma in their families or communities. Social and cultural views affect family support and influence AGYW's choices regarding abortion. Some participants recounted their parents' initial reactions to their pregnancy considering different social contexts. As one participant recalled, her mother initially responded negatively to her pregnancy. However, as she had finished high school, her mother became more supportive:

I was 20 years old when I fell pregnant, and the baby was born when I was 21 years old…I told my sister first… Ai, she did not believe me because when I told her that I was laughing as I also did not believe it…Thereafter I told the father of the baby, and he agreed…My mom was still here, so I told my mom, when I told my mom, I still remember it was in the afternoon, she was angry, but the next day she was fine…She told me that at least I respected her by completing my matric and then had a baby thereafter, that is how she accepted it. (Thabo Mofutsanyana, FS—18–24 years)

One participant faced pressure from her mother to abort her pregnancy as she did not want to raise grandchildren, which left her feeling upset:

At home they did not like it, saying that I should abort the baby…My mother said she won't have a baby…and took me to the clinic to abort…They asked me since I am four weeks and above plus I have now a baby book. They told me that means I want the baby, I replied yes I do, it's then when they reprimanded my mother why is she doing this…I did not feel good because we never sat down, me and my mother, to talk (about the pregnancy). (Tshwane 1, GAU—AGYW 18–24 years).

When participants were asked what they would do if they found out they were pregnant, some of them mentioned “abortion” in the context of choosing to have one vs. not having one. One participant in the 15–17 years age group said that since she was still in school and her family was already struggling financially, if she tested positive for pregnancy, she would think about getting an abortion, but asserted that since she would be underage, it would be for her parents to decide:

If I were pregnant here at home, I would inform my parents…And my parents would decide what to do, but as for me I would not keep that pregnancy whilst underage…It was going to be a heavy burden for me…And I'm still a scholar, I am not yet working and have no money to buy clothes for such a baby…Honestly speaking in my case, they were not going to be able to care for a baby because we are a struggling family. (Thabo Mofutsanyana, FS—15–17 years)

Contrastingly, most participants were against having abortions for a few reasons. As one participant said, if she found out that she was pregnant, she would not consider having an abortion because of fears of potential health complications:

I would keep the baby…Abortion is not an option…Because of future complication. (Ehlanzeni, MP—18–24 years).

Some participants were against abortions based on moral grounds, believing that it amounted to “killing an innocent child”. One participant did not think about getting an abortion when she had her first child, and she would not consider getting one if she became pregnant again:

I will do what I did (in my previous pregnancy), I will not do abortion, killing an innocent child…My parents kept me and gave me love so why should I not do the same. (Ehlanzeni, MP—18–24 years)

Similarly, some participants stated that they would not consider getting an abortion because it was against their religious convictions:

I would keep the baby, because my religious belief doesn't, allow me to terminate…I wouldn't terminate. (Tshwane 1, GAU—18–24 years)

The narratives of participants who actively chose abortion revealed the structural, emotional and interpersonal factors that shaped their experiences with abortion after confirming their pregnancy. One participant sought an illegal abortion due to being beyond the legal limit for the procedure, leading to hospitalisation after adverse effects from an abortion drug obtained through a Facebook advertisement. Despite initial community stigma and family tensions, the birth of a healthy baby ultimately restored family harmony:

The doctor had already known what I have done, and had already told my mother and my father about what I have done…I confessed about the whole truth I how I got the pill on Facebook ad, the doctor told me about how I put myself on danger buy doing that. I was told to visit the hospital every two weeks because my baby was high risk. I Apologized…The baby is fine and normal, nothing happened to the baby…I was embarrassed because everyone in my community had known that I wanted to do an abortion. My father was not speaking to me and my mother was calling me a liar and a killer, she said I am capable of killing someone, I should have been open with her and I then apologized. We were not on speaking terms until the baby was born, and he was happy and things are okay now. (Zululand, KZN—18–24 years)

Another participant had an abortion but was disappointed by the lack of compassion from the clinic staff. It was particularly challenging for her to openly request an abortion as it was her first time, considering she had given birth to her first baby shortly before falling pregnant again:

I actually had a termination of pregnancy, that happened uhm yes…the lady was being like whatever in a certain way…I felt very disappointed, because like it said in the previous interview the lady wasn't quite accommodating to me as I would have felt like at any other hospital or something…Uhm, basically telling them that I am coming for an abortion, that was like very difficult for me because its not everyday that I would walk into a hospital and just tell someone I am coming for an abortion also. I was nineteen. Nineteen yes and that was like uhm just say six seven months after I had my first baby. (Cape Town, WC—18–24 years)

Another participant expressed feelings of uncertainty regarding the information and expectations surrounding abortion. She described significant emotions post-abortion, including sadness, anger, and regret, identifying depression as a prominent feeling:

So I was scared, getting information and feedback that, “This is what you have to sign, this is what you have to take, this is what you have to do and what-not.” Yes…No. […] I thought I was depressed, very much sad, angry, ja [yes] very much depressed more than anything. Regret, even. (Tshwane 1, GAU—18–24 years).

One participant felt unprepared to raise another baby and decided to get an abortion. After the abortion, she was relieved to no longer be pregnant and experienced effective treatment during the procedure. She confided in her aunt, who supported her decision to abort, emphasising that with one child already, she could not manage another child at her age:

I was not ready…I got the pamphlet that they were distributing…I was happy that I was no longer pregnant…They treated me well…I talked to my aunt…She encouraged me to continue with an abortion because I already had a child, so she said I can't have two children at my age. (Ehlanzeni, MP—18–24 years).

## 4 Discussion

The AGYW pregnancy experience is primarily shaped by the mother's and unborn child's health, influenced by support from family, their partner, and non-judgmental HCW, and access to routine ANC. South African AGYW face societal, religious, and cultural expectations, which affect their access to pregnancy-related services. Our study found that support from both internal and external networks contributed to a positive pregnancy experience, with adequate emotional, financial, social, and health consultation support reducing health issues. Our study provides valuable insights through the inclusion of the perspectives of the impact of pregnancy on the lives of AGYW in South Africa, and the influence of support from family, their partners and healthcare workers on pregnancy experiences.

### 4.1 Support during pregnancy

This study found that pregnant AGYW's willingness to seek medical care, including ANC, was shaped by the support they received from family, partners, and HCWs. Family support helped to prevent emotional isolation and encouraged independent choices. Negative reactions to unplanned pregnancies often stem from fears of partner or family rejection due to gender and cultural norms. Most participants disclosed their pregnancies to their mothers but not their fathers. The level of support, thus, experienced by AGYW during and after pregnancy by their internal networks contributed to the experience of pregnancy for AGYW either positively or negatively (18, 30).

#### 4.1.1 Support from parents/caregivers

Our findings indicated that support from family members, particularly mothers, and frequent clinic visits played a crucial role in helping AGYW manage their health during pregnancy. This aligned with previous research (18), which found that after disclosing pregnancies, many young women received assistance from parents and caregivers, enabling them to care for their infants and continue their education. This support was especially vital for those still in school or pursuing higher education, reflecting the importance of familial backing for pregnancy AGYW.

Our findings also resonated with prior studies (18) who reported that fathers tended to be indifferent to their daughters' pregnancy-related stress, often resulting in strained relationships. This was consistent with broader literature indicating that mothers typically bear the greater responsibility for sexual education and communication within the family, despite fathers' expressed desire for more involvement (31). However, as highlighted by Duby et al. (32), mothers frequently felt discomfort, lack of confidence, or fear encouraging sexual behaviour when discussing these topics with their daughters. Despite these challenges, the present study's narratives suggested that most mothers ultimately provided ongoing support to their daughters during and after childbirth, reinforcing the critical role of maternal support.

#### 4.1.2 Support from partners

Our findings showed that when AGYW included the child's father during pregnancy, they benefitted from essential emotional and financial support, which contributed to heightened confidence and a greater sense of security. Partner involvement was especially significant in facilitating the process of disclosing pregnancy to family members and played a vital role in supporting AGYW's overall well-being. This observation was consistent with the findings of Karlström, Nystedt and Hildingsson (30), who emphasised the importance of the relationship between women and their child's father, noting it as a key factor in women's experiences during pregnancy.

Notably, men's active engagement in pregnancy was associated with a reduction in adverse outcomes, such as maternal and neonatal mortality, and unsafe abortions (33). By linking the data to these studies, partner involvement not only provided direct support but also contributed to positive health outcomes for both mother and child, reinforcing the critical natural supportive relationships during pregnancy.

#### 4.1.3 Impact of lack of support during pregnancy

AGYW experiences revealed how disclosure decisions were shaped by fear, anticipated stigma, and the availability—or absence—of supportive relationships. The difficulty many participants faced in deciding whom to inform about their pregnancy underscored the weight of social expectations on young women. Pregnancy outside of socially acceptable contexts becomes a moment of vulnerability (6). The hesitation to disclose their pregnancy suggested that AGYW anticipated judgment rather than support. The participant who chose not to inform her family illustrated how silence becomes a protective mechanism when emotional safety is uncertain.

A recurring theme was a fear of disappointing parents, reflecting deeply internalised norms around respectability, responsibility, and the moral expectations placed on young women. The possibility of parental rejection—whether emotional, financial, or physical—added another layer of risk. For some participants, this fear was so profound that it influenced their reproductive decisions, including opting for abortion. This finding suggested that family dynamics and perceived parental attitudes played a significant role in shaping AGYW's pregnancy journeys (34). Our findings also revealed how partner relationships could shift during pregnancy. The participant whose partner began making excuses when asked for financial support illustrates a broader pattern: pregnancy can expose the fragility of romantic relationships among young people. When partners withdraw support, AGYW are left to handle the emotional and maternal burdens alone (6, 35).

### 4.2 Mental health and pregnancy

Participants' narratives revealed the emotional toll of difficult pregnancy experiences, including premature births, miscarriages, and stillbirths. These events are not only physically taxing but also psychologically traumatic. The accounts of declining health, emotional distress, and symptoms of depression highlighted how adverse pregnancy outcomes can have lasting mental health consequences. Importantly, the presence of supportive figures—such as social workers, family members, and friends—played a crucial role in helping participants process their grief and begin healing. This underscores the value of accessible psychosocial support for AGYW experiencing reproductive loss or complications (36, 37).

Our findings showed that when AGYW received empathetic, consistent support, it significantly buffered the emotional impact of traumatic pregnancy experiences. Social workers emerged as key sources of emotional validation and guidance. Although our study did not detail the specific pathways through which AGYW initially accessed social workers under these circumstances, prior literature indicates that, in South Africa, healthcare professionals—such as midwives, nurses, and physicians—tend to initiate referrals for social work intervention based on their clinical observations during and following childbirth (38). Their involvement helped participants articulate their feelings, navigate grief, and regain a sense of stability (39). This suggested that formal support structures within schools and community healthcare facilities could serve as critical lifelines for young women facing reproductive challenges.

Stigma surrounding AGYW pregnancy remained a significant issue shaping their experiences. Participants described how fear of judgment—especially from elders—discouraged pregnant AGYW from seeking timely healthcare. This stigma not only affected emotional well-being but also had direct implications for maternal and foetal health. Delayed ANC, avoidance of clinics, and attempts to conceal pregnancy until it became physically obvious all reflect the ways stigma drove pregnant AGYW into secrecy, which was consistent with prior studies (40). These forms of behaviour increased the risk of unmanaged complications and reinforced cycles of shame and isolation. The reluctance to seek care is not simply a matter of personal choice; it is shaped by community norms and the perceived consequences of being visibly pregnant at a young age.

### 4.3 Health service experiences during pregnancy

The relationship between AGYW, clinic staff, and service organisation had the potential to impact access to pregnancy-related services. Pregnant AGYW were more likely to seek medical attention when treated well by HCW during ANC, as interactions with HCW could motivate or discourage them from attending ANC (9); the latter were more commonly mentioned by participants in our study. However, issues like unfavourable treatment from staff and inadequate management of grievance reporting systems exacerbated the negative patient-provider relationship.

#### 4.3.1 Health service access during pregnancy

Our findings revealed a mixed landscape of ANC experiences among AGYW, highlighting both positive encounters with the healthcare system and persistent gaps that undermined equitable, youth-friendly care. Some participants described their pregnancy journeys as relatively smooth, with accessible and supportive healthcare workers. These accounts demonstrated that when clinics functioned effectively and staff adopted compassionate, non-judgmental approaches, AGYW received the care they need without significant barriers. Emotional support from HCWs was particularly valued, suggesting that relational aspects of care were as important as clinical services (41). These positive experiences illustrated the potential of public health facilities to provide high quality, youth-friendly ANC when systems and staff attitudes aligned.

Despite these positive accounts, a subset of participants reported unfavourable interactions with ANC services. The mistreatment described—such as dismissive attitudes, judgmental comments, and lack of emotional support—reflected age-related stigma within healthcare settings (33). The participant who was told she was “too young to be pregnant” highlighted how moral judgments (42) could overshadow professional responsibilities. Such experiences could erode trust in the healthcare system and discourage AGYW from seeking timely care (4), ultimately affecting maternal and foetal health outcomes. Participants in the study by Young et al. (43) reported similar negative experiences with HCWs, however, the healthcare benefits of ANC outweighed any negative interactions, which differed from participant experiences in our study.

The perception that complaints from pregnant AGYW were not taken seriously pointed to systemic weaknesses in accountability and patient protection. When young women felt that their concerns were ignored, it reinforced power imbalances between patients and providers, which signaled that mistreatment was tolerated. These findings were consistent with Ngoma-Hazemba et al. (44), who reported that adolescents in Zambia encountered barriers within health services, including a lack of policies and procedures to safeguard their rights to information and care, reiterating previous research that highlighted the importance of positive patient-provider relationships (45). This dynamic can further silence AGYW, who may already feel vulnerable because of their age, social position, or pregnancy status (6, 46).

Participants also described logistical and structural barriers that compounded their negative experiences. Being turned away for arriving late, asked to return on another day, or waiting long hours only to be denied care reflects inefficiencies that disproportionately affect AGYW (43)—many of whom rely on limited financial resources and must travel long distances (45, 47). These experiences not only disrupted continuity of care but also created emotional distress (9, 43), as participants expressed sadness and discouragement at being treated dismissively.

The coexistence of positive and negative experiences suggested that ANC quality was inconsistent across facilities, staff members, and even individual encounters in our study. This inconsistency could create uncertainty for AGYW, who may not know whether they will be supported or stigmatised when seeking care. Such unpredictability could also influence health-seeking behaviours, potentially leading to delayed ANC initiation or reduced engagement with services (46).

#### 4.3.2 Views on accessibility of healthcare services

The organisation and operations of healthcare facilities emerged as a central factor influencing whether AGYW could meaningfully access ANC. Participants' accounts showed that proximity to clinics played a significant role: when services were located within or near their communities, AGYW found it easier to attend appointments and follow referral pathways. Conversely, when clinics were located farther away, the cost and time required for travel became substantial obstacles. For AGYW—who often have limited financial independence—these barriers could be prohibitive. These findings aligned with another study which documented how logistical and financial barriers discouraged regular healthcare use for young mothers (45). The need to spend money on transport to attend appointments underscored how structural constraints could undermine consistent ANC attendance.

The findings also pointed to the significance of the relationship between AGYW and clinic staff. When staff were approachable, supportive, and efficient in organising services, AGYW experienced fewer barriers to care (8, 15). However, when staff attitudes were judgmental or dismissive, the organisation of services became less effective, even if the facility was physically accessible. This reinforced the idea that accessibility was not only about infrastructure but also the quality of interpersonal interactions within the health system.

Fear of criticism for becoming pregnant at a young age emerged as a significant deterrent to seeking care. This stigma—rooted in community norms and reinforced by adults, including HCWs—could discourage AGYW from initiating ANC early or attending consistently. This was consistent with the findings of Erasmus et al. (8), which found that gendered sociocultural norms and practices stigmatised pregnancy among AGYW and created a culture of shame and non-disclosure, which significantly impeded access to ANC. Even when services were geographically accessible (45), the emotional cost of anticipated judgement could outweigh the perceived benefits of care. This dynamic illustrated how social barriers could be just as limiting as structural ones.

The findings showed that structural, financial, and social barriers do not operate in isolation. Instead, they intersect to shape AGYW's health-seeking behaviours (15). For example, a young woman who must travel far to a clinic may already be hesitant because of stigma, and the added financial burden may further discourage her from attending, contributing to delayed ANC initiation, missed appointments, and poorer maternal health outcomes. Overall, our findings and existing literature suggest that improving organisational factors and reducing external barriers are essential for enhancing AGYW's healthcare engagement.

### 4.4 Social and cultural contexts affecting pregnancy experiences

Our study findings revealed that social and cultural attitudes within communities significantly shaped maternal behaviour during pregnancy. AGYW who became pregnant faced stigma, which not only impacted on their mental health but also influenced family support and their decisions about health care and abortions. Participant narratives from the study illustrate the complexity of these influences.

Participants' hypothetical decisions regarding abortion were shaped not only by practical factors such as financial circumstances, school enrolment, and household dependents, but also by social and cultural stigma surrounding abortion. For two of our participants, the decision to terminate a pregnancy would be made by parents or caregivers, where the assumption of control over pregnancy-related choices by parents could be attributed to their child's age and familial circumstances in the household. This reflected both their limited autonomy and the influence of familial and societal attitudes towards abortion. The literature suggested that stigma could manifest in the form of parental control or pressure, as families sought to avoid shame or negative judgement from their communities (9, 48). Conversely, for AGYW who refused abortion, decisions were guided by religious beliefs, personal convictions, and concerns about future health implications—frequently reinforced by community norms and the prevailing stigma attached to abortion. This stigma often created a sense of fear, secrecy, and moral judgment, which may profoundly impact AGYW's sense of agency, emotional well-being, and their ability to make autonomous decisions about their pregnancies (48, 49).

Judgemental attitudes of both community members, who often viewed AGYW as transgressing social norms by engaging in sexual activity, served as additional obstacles to services such as abortion care. One participant's resort to an illegal abortion underscored the consequences of restrictive legal limits and limited access to safe services. Her experience—purchasing an abortion drug through a Facebook advertisement and subsequently being hospitalised—illustrated how legal constraints can push women toward unsafe methods (49). It also highlighted the role of community stigma; initial tensions and judgment from her social environment intensified her vulnerability. This notion showed similarities with the findings of Zia et al. (10), suggesting that AGYW feel stigmatised after having an abortion because they internalise societal standards. However, the eventual birth of a healthy baby served as a catalyst for restoring family harmony, suggesting that social acceptance could be conditional and deeply tied to reproductive outcomes. According to one study on attitudes towards various cultural contexts (50), negative and judgmental views about abortion may result in stigmatisation of women receiving care and create obstacles in obtaining necessary abortion care. This observation, as seen in this experience of abortion, revealed how societal and cultural norms around motherhood shape women's experiences before and after pregnancy.

Another participant's disappointment with the lack of compassion from clinic staff pointed to gaps in the quality of abortion care. Her difficulty in openly requesting the procedure—especially after recently giving birth—suggested that internalised shame and fear of judgment could be exacerbated by unsupportive healthcare environments (42, 48). The accounts also revealed the emotional complexity surrounding abortion. One participant described profound feelings of sadness, anger, regret and depression, reflecting how abortion could intersect with broader psychological and social stressors. As one review of the mental health implications of abortions explains, a woman's psychological reaction after an abortion may be influenced by past trauma and mental health diagnosis (51). Additionally, her uncertainty about the information provided suggested that inadequate counselling or unclear communication may have contributed to emotional distress. Similar to the findings of Sewpaul et al. (9), the lack of information regarding procedures from HCWs underscored the need for comprehensive pre- and post-abortion support.

In contrast, another participant's experiences demonstrated that abortion could also bring relief and a sense of regained control. Feeling unprepared for another child, she was supported by her aunt and received respectful treatment during the procedure. Her narrative highlighted how social support (37) and positive clinical interactions could mitigate emotional strain and affirm reproductive autonomy (52).

Across these experiences of abortion and decisions in the choice of abortion, the presence—or absence—of supportive individuals significantly shaped participants' experiences. Supportive family members provided emotional validation and practical assurance. Conversely, stigma or judgment from others intensified distress. This suggested that social networks play a crucial role in shaping women's emotional well-being during abortion-related decision-making.

### 4.5 Strengths and limitations of the study

A core strength of the study was the insights into complex behaviour and motivations related to support-seeking, health-seeking, and abortion decisions. It also explored pregnancy views and experiences, providing valuable insights into perceptions and narratives of AGYW living in communities where SRH intervention programmes were implemented.

The study, however, faced technical challenges in remote data collection, including poor internet connectivity, network coverage, and electricity outages. These issues hindered interviewers' ability to screen participants, conduct interviews, and record high-quality audio, often causing interviews to be paused or rescheduled. Enrolling participants in the qualitative study also faced some challenges. There was a high volume of unsuccessful calls, with interviewers reporting that many attempted screening calls went straight to voicemail. Many AGYW shared phones with family members, making confidential interviews challenging and time-consuming. The lack of non-verbal cues also made it difficult to build rapport and assess the participants' engagement effectively.

The lack of adequate regional and age-based disparities may also restrict the generalisability of the study's findings. It was challenging to generalise the findings broadly since most of the participants were in the Western Cape province, where their experiences and perspectives were likely similar and might not fairly represent the larger population in other areas. Furthermore, because more young women than adolescent girls participated in the interviews, it was difficult to ascertain whether the experiences and perspectives of pregnancy and related care were unique to that narrow age range or if they applied to people with different life experiences or to people of all ages. The underrepresentation of adolescent girls may result from cultural or social barriers and parental consent challenges that limited their study participation. Nevertheless, the evidence presented in this study is still helpful for comprehending this social phenomenon in the South African context, especially in these regions where SRH intervention programmes, such as My Journey, are offered.

Lastly, participants may have been influenced by social desirability bias, particularly regarding sensitive topics like mental health and pregnancy, possibly resulting in underreporting negative experiences. Additionally, recall memory bias could affect the accuracy of their reports, as recollections of earlier pregnancy experiences might be flawed. However, to mitigate these potential issues, open-ended questions were included in the semi-structured interview guide to encourage participants to share their perspectives and experiences freely, while leading questions were avoided to prevent prompting specific responses.

### 4.6 Conclusions and implications of the study

The findings of this study shed light on AGYW pregnancy and ANC service experiences. The description of viewpoints provides important insights into the interpersonal aspects influencing the experience of pregnancy in not only adolescent girls but also that of young women in both rural and urban settings—an area that has not been thoroughly documented in previous studies. These findings provide critical evidence which can help to inform the implementation of responsive, appropriate, and accessible youth-friendly SRH services and interventions in communities with reported high rates of AGYW pregnancies and births. To enable AGYW to make safe, knowledgeable, independent, and responsible decisions and behaviours regarding their own sexual and reproductive health, efforts must be taken to establish these foundations (42).

These findings also indicate that policymakers and healthcare professionals should priorities the training of HCWs and support staff who interact with AGYW (43). This training should emphasise sensitivity, non-judgmental attitudes, and respectful communication to ensure that AGYW feel welcomed and supported in healthcare settings. Hospitals and clinics should regularly review their policies and practices, aiming to eliminate punitive or discriminatory approaches and instead promote youth-friendly spaces where AGYW can access care and disclose pregnancies without fear of stigma. Strengthening patient-provider communication through structured guidelines and feedback, as well as offering culturally appropriate strategies for discussing sensitive topics, will also enhance the quality of care. Mental health screening and support should also be seamlessly incorporated into routine antenatal and postnatal care (34) with clear referral pathways to counselling or psychological services for those at risk of depression, anxiety or self-harm—conditions more likely in the absence of strong social support networks (6, 53).

Lastly, interventions must actively involve families and partners in the care process. Providing educational sessions and support groups for parents and partners—especially for mothers and fathers eager to participate more fully—can foster open communication, emotional support, and a positive environment for AGYW. Community-based research should be conducted to better understand sociocultural and traditional factors influencing care-seeking behaviour (24), allowing for the development of culturally-sensitive interventions that encourage early initiation of ANC. Policymakers should promote integrated frameworks linking health, education, and social support services, ensuring that young mothers can continue their education and experience improved health outcomes during and after pregnancy.

## Data Availability

The raw data supporting the conclusions of this article will be made available by the authors, without undue reservation.
